# A new paradigm: innate immune sensing of viruses via the unfolded protein response

**DOI:** 10.3389/fmicb.2014.00222

**Published:** 2014-05-16

**Authors:** Judith A. Smith

**Affiliations:** Department of Pediatrics, University of Wisconsin School of Medicine and Public HealthMadison, WI, USA

**Keywords:** unfolded protein response, viruses, type I IFN, innate immunity, XBP1, ER stress, pattern recognition receptors

## Abstract

The immune system depends upon combinations of signals to mount appropriate responses: pathogen specific signals in the context of co-stimulatory “danger” signals drive immune strength and accuracy. Viral infections trigger anti-viral type I interferon (IFN) responses by stimulating endosomal and cytosolic pattern recognition receptors (PRRs). However, viruses have also evolved many strategies to counteract IFN responses. Are there intracellular danger signals that enhance immune responses to viruses? During infection, viruses place a heavy demand on the protein folding machinery of the host endoplasmic reticulum (ER). To survive ER stress, host cells mount an unfolded protein response (UPR) to decrease ER protein load and enhance protein-folding capacity. Viruses also directly elicit the UPR to enhance their replication. Increasing evidence supports an intersection between the host UPR and inflammation, in particular the production of pro-inflammatory cytokines and type I IFN. The UPR directly activates pro-inflammatory cytokine transcription factors and dramatically enhances cytokine production in response to viral PRR engagement. Additionally, viral PRR engagement may stimulate specific pathways within the UPR to enhance cytokine production. Through these mechanisms, viral detection via the UPR and inflammatory cytokine production are intertwined. Consequently, the UPR response is perfectly poised to act as an infection-triggered “danger” signal. The UPR may serve as an internal “co-stimulatory” signal that (1) provides specificity and (2) critically augments responses to overcome viral subterfuge. Further work is needed to test this hypothesis during viral infections.

## INTRODUCTION: TUNING AN APPROPRIATE IMMUNE RESPONSE

Inappropriate activation of the immune system, as evident by toxic shock and autoimmune diseases, reveals an incredibly potent force that can wreak havoc on the human body. Thus multiple safeguards are in place to ensure self-tolerance, including activation induced cell death, anergy, ignorance, regulatory cytokine networks, and T-regulatory cells (Walker and Abbas, 2002; Bluestone and Bour-Jordan, 2012). However, in the face of a foreign invader, the immune system must respond quickly and dynamically. Much investigative emphasis has been placed on combinations of signals that ramp up the adaptive immune response to infectious challenges. Conserved structural components of the pathogens provide essential immune stimulatory signals. These pathogen-associated molecular patterns (PAMPs; e.g., lipopolysaccharide (LPS), peptidoglycan, flagellin, zymosan) are recognized by cell surface pattern recognition receptors (PRRs) on immune cells. One class of PRRs, the Toll-like receptor (TLR) family, responds to a broad spectrum of pathogens. Endogenous products produced during concomitant tissue destruction during infection, so called “danger associated molecular patterns” (DAMPs) also stimulate PRRs (Matzinger, 1994; Bianchi, 2007; Tang et al., 2012). Engagement of PRRs on macrophages and dendritic cells enhances antigen presentation, expression of T cell co-stimulatory molecules, and provides an inflammatory cytokine milieu. Through these combinations of stimuli, cells are poised to respond appropriately to external threats.

However, not all immune stimuli remain extracellular. Also, infected cells must cope until an effective adaptive immune response can be mobilized. Intracellular pathogens such as viruses excite immune responses by triggering endosomal and cytosolic PRRs. Host cells detect viral dsRNA via endosomally localized TLR3, cytosolic RNA-helicases such as retinoic acid inducible gene 1 (RIG-I) and melanoma differentiation associated 5 (MDA-5), and interferon induced sensors such as protein kinase R (PKR). Additionally, endosomal TLR7/8 responds to ssRNA, TLR9 senses CpG oligodinucleotides, and a variety of cytosolic PRRs (e.g., DAI, AIM2 etc.) recognize DNA (Thompson et al., 2011; Goubau et al., 2013; Szabo and Rajnavolgyi, 2013). Early during viral infection, engagement of PRRs leads to the transcription of type I IFN genes that are regulated by the transcription factor interferon regulatory factor 3 (IRF3), including IFN-β and limited species of IFN-α (Hiscott, 2007). This initial wave of IFN serves as an “alarm signal”: binding of early IFN to the type I IFN receptor (IFNAR) triggers Janus kinase 1/tyrosine kinase 2 – signal transducers and activators of transcription 1/2 (JAK1/Tyk2–STAT1/2) signaling, and thus an anti-viral transcriptional program (Levy et al., 2003). IFNAR-regulated genes include IRF7, which induces transcription of multiple IFN-α genes, the dsRNA sensor PKR, and other interferon-stimulated genes (ISGs) that enhance viral recognition and interfere with multiple steps of viral replication (Sato et al., 2000). This PRR-elicited anti-viral transcriptional program plays a critical role in controlling infection.

There are several challenges to the generation of an effective anti-viral program following PRR engagement, including specificity, strength of signal, and viral sabotage. It is not completely clear how the host differentiates between ssRNA, dsRNA, and dsDNA of host and pathogen origin. MDA5 can distinguish a ribose 2′ *O*-methylation found on host mRNA (Zust et al., 2011). However, NS5 of flaviviruses such as Dengue virus (DENV) cap viral RNA with 2′ *O*-methylation to evade detection (Dong et al., 2012). Another potential mechanism to resolve host and pathogen resides in the compartmentalization of host nucleic acids and corresponding PRRs. Stimulation of PRRs with purified agonists alone, such as LPS or the synthetic dsRNA polyI:C, leads to weak, barely detectable amounts of IFN in macrophages (Smith et al., 2008). Engagement of multiple types of PRRs by different motifs on a complex pathogen may be required to synergize (Nasirudeen et al., 2011; Szabo and Rajnavolgyi, 2013). Finally, viruses have evolved numerous strategies to combat IFN signaling at multiple levels, from production of early IFN to IFNAR signaling. For instance, Coronavirus antagonizes a molecule in the DNA-sensing pathway, Stimulator of Interferon Gene (STING/MITA) by disrupting its association with the IRF3-activating kinase tank binding kinase 1 (TBK1)/IKKε (Ishikawa et al., 2009; Sun et al., 2012). Respiratory syncytial virus (RSV) disrupts association between IRF3 and the transcriptional co-activator CREB binding protein (CBP)/p300 (Ren et al., 2011). Vesicular stomatitis virus (VSV) and Hepatitis C virus (HCV) targets the IFNAR receptor for degradation (Liu et al., 2009). DENV cleaves STING, blocks Tyk2 phosphorylation, impairs STAT1 phosphorylation, and targets STAT2 for proteosomal degradation (Green et al., 2014). Paramyxovirus induces degradation of STAT1 and STAT2 (Horvath, 2004). In the face of all these challenges to the PRR-induced anti-viral program, might there also be *intracellular* co-stimulatory or “danger” signals that provide context and critically augment the immune response to ensure success?

## VIRUSES AND ER STRESS

Production of high numbers of new virions within a host cell places inordinate stress on the protein folding machinery of the host endoplasmic reticulum (ER). To survive ER stress, the host cell mounts a response known as the “Unfolded Protein Response” or UPR (Schroder and Kaufman, 2005). In the co-evolutionary dance between host and invader, viruses have manipulated this host stress response to enhance viral reproduction. However, in the past decade it has become apparent that the UPR, or specific pathways within the UPR, can promote inflammatory cytokine production. Thus, the UPR may be poised to serve as an internal “danger” signal, complementing PRRs in alerting a cell to invasion and boosting subsequent immune responses (Dalod and Pierre, 2011). The case for UPR as viral-triggered immune stress signal will be reviewed below.

### UPR PATHWAYS

**FIGURE 1:**
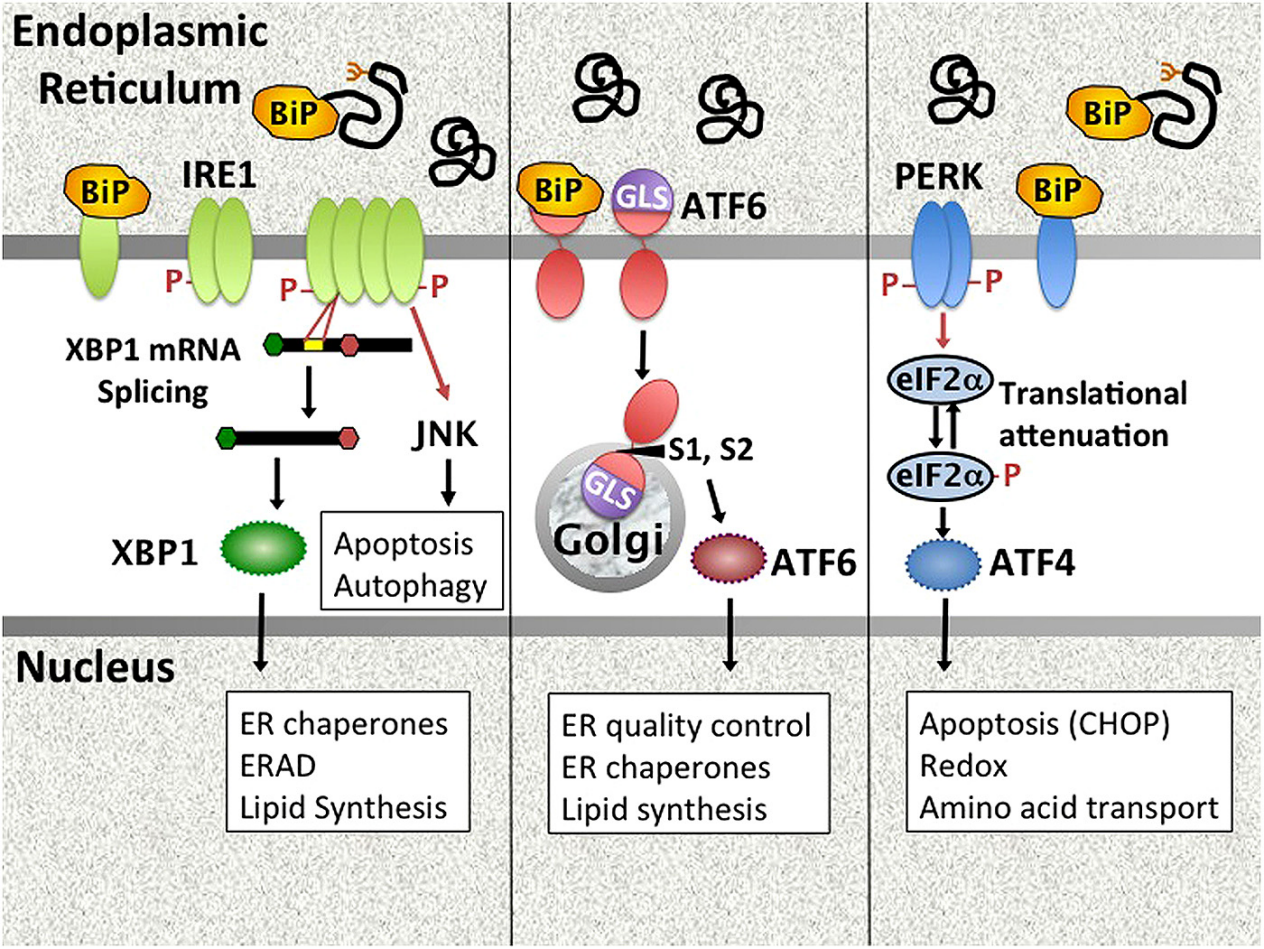
**Mammalian UPR pathways.** The UPR encompasses signaling pathways triggered by the activation of ER stress transducers IRE1, ATF6, and PERK. In unstressed cells, these molecules associate with the folding chaperone BiP. Upon accumulation of unfolded proteins in the ER, PERK, and IRE1 release BiP and oligomerize. IRE1 is both a kinase that phosphorylates targets such as JNK, and an endonuclease that splices 26bp from the XBP1 mRNA, removing a premature stop codon. Dissociation of ATF6 from BiP uncovers a Golgi localization signal. ATF6 traffics to the Golgi, where site-specific proteases (S1, S2) cleave it to an active transcription factor. PERK phosphorylates eIF2α, resulting in global translational attenuation apart from select open reading frames (e.g., ATF4). UPR gene targets (e.g., CHOP) and UPR regulated cellular processes are in boxes. ERAD = ER associated degradation. GLS = Golgi localization signal.

The ER controls vital cell functions including protein folding, post-translational modifications, calcium storage, and lipid membrane biosynthesis. Physiologic stresses (increased protein secretion, misfolding proteins) and environmental perturbations (e.g., nutrient starvation, calcium dysregulation, hypoxia etc.) may derail ER function. The UPR is an evolutionarily conserved stress response that maintains ER homeostasis (Hetz et al., 2011; Walter and Ron, 2011). In the unstressed state, UPR initiation molecules residing in the ER membrane are held in check through association with the folding chaperone BiP/GRP78. During ER stress, BiP is released from three primary stress-transducers, activating transcription factor (ATF6), inositol requiring kinase 1 (IRE1), and PKR-like endoplasmic reticulum kinase (PERK), thus activating downstream signaling pathways (**Figure 1**). This activation step may involve multiple potential mechanisms, including competitive sequestration of BiP by misfolded proteins (PERK and IRE1), direct sensing of misfolded proteins by the IRE1 (and by analogy PERK) luminal domains, as well as active dissociation of BiP from ATF6 through an undefined mechanism (Ron and Walter, 2007; Shen et al., 2005).

(1) Dissociation of BiP from ATF6 uncovers a Golgi localization signal, enabling egress from the ER. Upon transit to the Golgi, site-specific proteases (S1P and S2P) cleave ATF6 to release the active transcription factor, which then induces UPR target genes (Adachi et al., 2008). (2) IRE1 has dual functions as both kinase and endonuclease (Hetz et al., 2011). The only known specific mRNA target for the endonuclease function is the transcription factor X-box binding protein 1 (XBP1). IRE1 cleaves 26bp from the XBP1 mRNA, thus removing a premature stop codon. The unconventionally spliced XBP1 mRNA encodes the full length XBP1 containing a transcriptional transactivation domain. Coordinately and independently ATF6 and XBP1 regulate chaperones and other proteins involved in folding and ER-associated protein degradation (ERAD; Lee et al., 2003; Adachi et al., 2008). XBP1 also critically regulates lipid synthesis, promoting expansion of the ER (Sriburi et al., 2004). In addition to XBP1 splicing, IRE1 endonuclease activity also regulates multiple microRNAs, including miR-17, thus relieving translational repression of molecules involved in apoptosis such as Caspase-2 (Upton et al., 2012). Finally, IRE1 has a non-specific nuclease activity that degrades ER membrane associated mRNAs encoding mostly secretory proteins in a process known as regulated IRE1 dependent decay (RIDD; Hollien and Weissman, 2006; Hollien et al., 2009). Related to its kinase activity, IRE1 forms a multi-molecular complex (“UPRosome”) with TNF receptor-associated factor 2 (TRAF2) and apoptosis signal-regulating kinase 1 (ASK1) that triggers multiple signaling pathways and cellular processes, including jun N-terminal kinase (JNK) signaling, autophagy, and the regulation of apoptosis vs. survival (Woehlbier and Hetz, 2011). (3) Upon release of BiP, PERK dimerizes, and auto-transphosphorylates to activate its kinase activity. PERK in turn phosphorylates eIF2α, resulting in global translational attenuation apart from select open reading frames. One of the primary targets for this selective translation is the transcription factor ATF4, which regulates amino acid transport, protection against oxidative stress, and apoptosis via CHOP (Walter and Ron, 2011). ATF4 induced growth arrest and DNA damage inducible 34 (GADD34) associates with protein phosphatase 1 to mediate dephosphorylation of eIF2α, thus turning off the PERK pathway in a negative feedback loop. As another example of cross talk between pathways, XBP1-induced p58^ipk^ binds PERK and inhibits its kinase activity (Lee et al., 2003; van Huizen et al., 2003). Translational attenuation decreases ER client load, but the transitory duration ensures cell survival.

Together, these three primary effector-dependent biochemical pathways induce a gene transcriptional program that enables cells to cope with stress by enhancing protein folding and decreasing protein load in the ER. In addition to regulating protein synthesis, the UPR exerts a profound effect on multiple cellular processes including autophagy, apoptosis, ER and Golgi biogenesis, Redox status, and lipid synthesis. If ER stress remains unresolved despite these adaptive measures, the UPR initiates apoptosis. Related to its role in supporting protein production, the UPR is physiologically active in highly secretory cells such as pancreatic acinar cells, hepatocytes, and Paneth cells (Lee et al., 2005; Kaser et al., 2010). However, the UPR also apparently plays a critical role in immune cell homeostasis, being required for plasma cell development from B-lymphocytes and the development and survival of both myeloid and plasmacytoid dendritic cells (Iwakoshi et al., 2003, 2007).

### VIRUSES AND THE UPR

In order to replicate, viruses must utilize host ER to produce greatly increased quantities of viral protein, inducing ER stress. Although the increased folding capacity of the UPR should benefit viruses, translational attenuation, ERAD, and host apoptosis could all potentially limit viral replication. Thus perhaps it is not surprising that many viruses have evolved strategies to manipulate different aspects of the host UPR (He, 2006). Viruses induce the UPR in various ways, including greatly increasing protein synthesis, elaboration of misfolded proteins (e.g., hemagglutinin) and direct interaction with BiP, as seen with the US11 protein of human cytomegalovirus (HCMV; Hurtley et al., 1989; He, 2006; Hegde et al., 2006). The extent of UPR induction varies between viruses and reports describing individual viruses have also varied over the years, complicating interpretation of the literature. For instance reports investigating HCV have commented on isolated ATF6 cleavage, ATF6, and XBP1 splicing (but inhibition of downstream XBP1 target induction), or induction of all three major arms of the UPR (Tardif et al., 2002, 2004; Ke and Chen, 2011; Merquiol et al., 2011). Some of these discrepancies may arise from investigations of individual viral protein vs. whole cell infections, as well as choice of host cell. Some viruses selectively induce parts of the UPR. For instance, HCMV US11 induces XBP1 splicing (without downstream EDEM induction) but does not lead to ATF6 cleavage (Isler et al., 2005). West Nile virus activates XBP1 and ATF6 but inhibits PERK activity (Ambrose and Mackenzie, 2011). Lymphocytic choriomeningitis virus selectively activates ATF6, but not PERK or IRE1 (Pasqual et al., 2011). Epstein Barr virus (EBV) appears to induce all three axes, with a feed forward loop of EBV LMP protein activating PERK and the PERK-dependent ATF4 inducing viral LMP (Lee and Sugden, 2008). Viruses may also activate different arms of the UPR at different times following infection. For instance, one report on DENV describes early PERK activation followed by inhibition, XBP1 induction mid-infection and ATF6 activation late in infection (Pena and Harris, 2011). In this case, CHOP induction did not lead to activation of caspases and apoptosis. PERK inhibition appears to be a common thread between different viruses. One of the most notable examples is the Herpes simplex virus (HSV) protein γ_1_34.5/ICP34.5 that acts analogously to the GADD34 target to relieve translational inhibition (He et al., 1997; Cheng et al., 2005). Induction of the UPR, or parts of the UPR, appears to be essential for promoting viral lifestyle. Consequently, blockade or knockdown of the UPR pathways adversely impact viral replication and increase cytopathic effects (Yu et al., 2006; Ke and Chen, 2011; Ambrose and Mackenzie, 2013).

The direct induction of the UPR by viral proteins, as well as the host response to increased protein load in the ER both position the UPR well to serve as an intracellular “danger signal” alerting the cell to infection. Interestingly, multiple UPR pathways appear to share evolutionary history with dedicated anti-viral pathways. PERK is evolutionarily related to the interferon induced PKR (as PERK’s name implies). PKR responds directly to dsRNA by phosphorylating eIF2α (analogously to PERK) in an effort to halt viral protein synthesis (He, 2006). GCN2, a third eIF2α kinase family member responsive to amino acid starvation is induced by Sindbis virus and inhibits replication (Berlanga et al., 2006). IRE1 is related to the anti-viral molecule RNAse-L both in structure and function (>40% similarity; Chakrabarti et al., 2011; Martinon and Glimcher, 2011). Like RNAse-L, the non-specific endonuclease activity of IRE1 generates small RNA species with 5′OH and cyclic 2′3′ phosphodiester 3′ ends that can be recognized by RIG-I (Cho et al., 2013). Thus perhaps it is not merely coincidence that the UPR should be engaged during viral infection. How then does this stress response interact with host immune, and more specifically anti-viral responses?

## THE INTERSECTION OF ER STRESS AND INFLAMMATION

Beyond its role in supporting immune cell development, the UPR has become increasingly implicated in various inflammatory conditions ranging from obesity and atherosclerosis to diabetes, neurodegenerative diseases, arthritis, and inflammatory bowel disease (Zhang and Kaufman, 2008; Wang and Kaufman, 2012; Claudio et al., 2013). Is the UPR an inflammatory instigator or byproduct of the inflammatory state (or both)?

### DIRECT INFLAMMATORY SIGNALING BY THE UPR

Over the past decade, it has become apparent that the UPR directly triggers inflammatory signal transduction pathways, including mitogen activated protein (MAP) kinase (ERK1/2, p38, and JNK) signaling, and activates key inflammatory transcription factors such as nuclear factor kappa-light chain enhancer of activated B cells (NF-κB; Zhang and Kaufman, 2008; Hotamisligil, 2010; Hasnain et al., 2012). In unstimulated cells, NF-κB family members (p50, p52, p62, RelB, and c-Rel) are sequestered in the cytoplasm by association with an inhibitory molecule inhibitor of κB (e.g., IκBα). Upon stimulation (e.g., PRR engagement), IκB kinase (IKK) phosphorylates IκBα, targeting it for ubiquitination and proteosomal degradation. Dissociation from IκBα allows NF-κB to transit to the nucleus where it can induce cytokines such as TNF-a and IL-6 (Hayden and Ghosh, 2008). In Li et al. (2005), reported that free cholesterol-induced MAP kinase signaling and NF-κB activation in macrophages required transit of the cholesterol to the ER and induction of ER stress. Other examples of non-infectious UPR-related inflammation have since been described: the oxidized phospholipid-stimulated UPR regulates cytokine production by human endothelial cells (Gargalovic et al., 2006). Pharmacologic agents that induce the UPR such as tunicamycin (N-linked glycosylation inhibitor) or thapsigargin (SERCA pump inhibitor) also stimulate low-level inflammatory cytokine production (e.g., IL-6; Martinon et al., 2010; Peters and Raghavan, 2011).

Multiple UPR pathways participate in NF-κB activation. In the free cholesterol-loaded macrophages, CHOP was apparently necessary for full induction of ERK1/2 phosphorylation and IL-6 production (Li et al., 2005). The mechanism remains unclear but may involve CHOP mediated antagonism of a negative regulator of NF-κB, peroxisome proliferator activator gamma (PPARγ; Park et al., 2010). PERK has also been proposed to activate NF-κB via translational attenuation, related to the relatively short half-life of IκBα compared to NF-κB (Deng et al., 2004). A second major arm of the UPR, stemming from IRE1 activation, also activates NF-κB. The IRE1–TRAF2 complex recruits IKK, potentially supporting basal activation of IKK, and thus contributing to NF-κB activation (Tam et al., 2012). IRE1–TRAF2 also stimulates JNK signaling via ASK1, leading to the activation of other cytokine-regulatory transcription factors belonging to the activator protein-1 (AP1) family (Urano et al., 2000; Nishitoh et al., 2002). Subtilase toxin induced activation of ATF6 also results in NF-κB activation, although the mechanism is not clear (Yamazaki et al., 2009). In addition to the three canonical UPR signaling pathways, ER stress (or ER “overload”) activates NF-κB through the generation of reactive oxygen species (ROS) and ER calcium release (Pahl and Baeuerle, 1997; Zhang and Kaufman, 2008). Mitochondria participate in this process, enhancing ROS production and ER calcium leak. In a positive feedback loop, the resulting inflammatory cytokines can trigger further ER stress through induction of more ROS (oxidative stress) and increasing release of calcium from the ER, interfering with chaperone function (Zhang and Kaufman, 2008).

Another potential feed-forward loop has been described in the liver. During ER stress, other molecules besides ATF6 undergo site directed proteolysis, including SREBP, CREBH, CREB4, Luman, and OASIS, possibly in a cell-specific, or context-specific manner (Bailey and O’Hare, 2007). In liver cells, the UPR leads to proteolytic activation of CREBH, which then induces key proteins in the acute phase response, serum amyloid protein and C-reactive protein (Zhang et al., 2006). Interestingly, TLR4 stimulation and inflammatory cytokines such as IL-6 can in turn induce the UPR in liver cells (Zhang et al., 2006). Hepatocytes are not unique in cytokine-triggered UPR activation: oligodendrocytes also exhibit modest BiP and CHOP upregulation upon stimulation with IFN-γ, consistent with an integrated stress response (Lin et al., 2005). Further, PERK activation may protect mature oligodendrocytes during demyelinating diseases (Lin et al., 2007).

### UPR–PRR SYNERGY AND IFN PRODUCTION

As this work on “sterile” inflammation occurred, other lines of investigation suggested a strong partnership between the UPR and infectious signals. In the field of rheumatology, it was noted that the molecule most strongly linked to spondyloarthritis, the MHC allele HLA-B27, misfolded, bound BiP excessively, and induced a UPR (Dangoria et al., 2002; Turner et al., 2005). Further, macrophages from diseased HLA-B27 transgenic rats showed transcriptomic evidence of both UPR (increased CHOP, BiP, Erp70, etc.) and IFN gene signature (Best5, MX1, Oas1, STAT2, Gbp2, IRF7, CXCL10, etc.; Turner et al., 2005). The association between IFN signature and UPR has been observed in other rheumatologic diseases, including systemic sclerosis and possibly specific types of myositis (Nagaraju et al., 2005; Gherardi, 2011; Lenna et al., 2013).

At first the link between UPR and type I IFN was not clear, as treatment of cells with UPR inducing pharmacologic agents such as tunicamycin and thapsigargin triggered virtually undetectable type I IFN (Smith et al., 2008). However, if cells undergoing an acute UPR were then treated with LPS (TLR4 agonist), poly I:C (TLR3) or transfected with poly I:C (MDA-5), the amount of IFN-β was augmented log-fold or more over the PRR agonist alone (Smith et al., 2008; Hu et al., 2011). In addition to IFN-β, the UPR augmented the specific production of other pro-inflammatory cytokines including IL-6, TNF-α, and IL-23, a cytokine implicated in the generation of pathogenic Th17 responses (Smith et al., 2008; DeLay et al., 2009; Martinon et al., 2010). It is not clear what portion of synergistic IFN-α or CXCL10 production reflected IFNAR signaling by primarily increased IFN-β(Smith et al., 2008; Hu et al., 2011). This phenomenon of synergy was not only observed upon pre-treatment with pharmacologic agents: macrophages from HLA-B27 transgenic rats also responded to TLR agonists such as LPS with greatly augmented IFN-β production (Smith et al., 2008). As another example, cells expressing the misfolding α-1 antitrypsin respond to LPS with greater cytokine production (Carroll et al., 2010). Further, relieving ER stress with agents such as chemical chaperones (e.g., 4-phenylbutyric acid, tauroursodeoxycholic acid), which aid in protein folding, can ameliorate LPS induced inflammation (Kim et al., 2013). Synergistic cytokine production has been observed in multiple culture cell types, as well as human macrophages, mouse macrophages, and dendritic cells (Smith et al., 2008; Hu et al., 2011). The synergism is inflammatory-mediator specific, in that it does not extend to all cytokines and chemokines. For instance, IL-1β and RANTES are not synergistically induced by TLR ligation and concomitant UPR (Smith et al., 2008; Martinon et al., 2010). PRR specificity may depend upon cell type: in macrophages, synergism occurs with stimulation of TLR2, TLR3, TLR4, and MDA-5 but not TLR7 and TLR9 (Smith et al., 2008; Martinon et al., 2010). However, in cells where these TLR7 and TLR9 are more prominently engaged, such as plasmacytoid dendritic cells, synergy is readily detected (Hu et al., 2011).

Synergism between environmental stimuli and ER stress made teleological sense for spondyloarthritis for several reasons: in the HLA-B27 rat model, disease does not develop in germ free animals, but reconstitution with limited colonic flora was sufficient, suggesting the need for an infectious trigger (Taurog et al., 1994). A specific type of spondyloarthritis, reactive arthritis, is classically initiated by Gram-negative infections of the gastrointestinal and genitourinary tract. Finally, spondyloarthritis patients often develop overt or subclinical inflammatory bowel disease, another manifestation linking UPR, microbial triggers, and inflammation (Mielants et al., 1988).

### MECHANISMS UNDERLYING UPR-PRR SYNERGY

IFN and inflammatory cytokine production is largely regulated by the nuclear availability and activation status of critical transcription factors. As described above, the ability of PERK-eIF2α and IRE1-kinase pathways to enhance the activation of NF-κB and AP1 should potentiate cytokine production by PRR agonists. However, it was not clear why the UPR–PRR interaction was synergistic rather than just additive. A requirement for cooperative transcription factor binding provides one possible explanation (Panne et al., 2007). Further investigation into the mechanisms underlying synergy revealed the involvement of other UPR pathways as well as more direct interaction between UPR-specific transcription factors and cytokine/IFN gene regulatory elements. Studies employing XBP1 gene knockdown, XBP1 deficient MEFs, and macrophages from conditional XBP1 knockout mice, together confirmed a critical role for the IRE1-dependent XBP1 transcription factor in synergistic cytokine production. XBP1 was essential for augmented IFN-β, ISG15, IL-6, TNF-α, and IL8 in response to combined ER stress and PRR signaling (Mielants et al., 1988; Smith et al., 2008; Martinon et al., 2010; Zeng et al., 2010). Indeed, XBP1 apparently plays a role in basal TLR-dependent cytokine production, even in the absence of UPR induction (discussed below). Chromatin immunoprecipitation (ChIP) studies revealed binding of XBP1 to IL-6 and TNF-α promoters as well as a TNF-α enhancer element (Martinon et al., 2010). A similar experimental approach revealed binding of another UPR-regulated transcription factor, CHOP to the IL-23 p19 promoter in dendritic cells (Goodall et al., 2010). The mechanism underlying synergistic IFN-β production, however, proved more elusive.

Regulation of the IFN-β encoding *ifnb1* gene has been intensively investigated and elegantly elucidated (Agalioti et al., 2000). The core *ifnb1* enhancer at -102 to -51 contains a series of tightly packed binding sites for members of the NF-κB family, AP1, IRF3, and IRF7 transcription factors (Panne et al., 2007). Following PRR stimulation, these transcription factors bind cooperatively to the site, forming an “enhanceosome” (Merika and Thanos, 2001). IRF3 associates with a histone acetyltransferase, CREB binding protein (CBP)/p300, thus bringing this transcriptional co-activator to the enhancer. Assembly of the enhanceosome results in sequential recruitment of chromatin modifying factors and basal transcription machinery. As a result of this process, an inhibitory nucleosome slides downstream, away from the TATA box, thus enabling transcription of IFN-β (Agalioti et al., 2000). Binding of IFN-β to the IFNAR receptor then results in new transcription of IRF7, which strengthens IFN-β transcription and leads to the production of multiple IFN-α genes and other ISGs (Sato et al., 2000).

IRF3 is absolutely required for initial LPS-induced IFN-β expression and early viral-induced IFN (Sato et al., 2000; Sakaguchi et al., 2003). Besides IFN-β and IFN-α4 (IFN-α1 in human), IRF3 regulates a subset of other ISGs, including ISG54, ISG56, and RANTES independently of IFNAR signaling (Grandvaux et al., 2002). IRF3 can also induce apoptosis through association with pro-apoptotic Bax (Chattopadhyay et al., 2010). In unstimulated cells, IRF3 resides in the nucleus. Upon stimulation, TBK1/IKKε family kinases phosphorylate IRF3 at multiple serines and threonines, enabling IRF3 dimerization, nuclear translocation, association with the CBP/p300 co-activator and DNA-binding activity (Hiscott, 2007). During viral infection, phosphorylation at IRF3 S385/S386 plays an important role in regulating phosphorylation in the 396–405 Ser/Thr cluster and strengthens the association with CBP (Chen et al., 2008). Partial phosphorylation will result in some of the activation steps leading from cytosol to nucleus, but will not permit full IRF3 transcriptional activity (Lin et al., 1999).

There are no XBP1 binding consensus sequences in the well-characterized *ifnb1* promoter/enhancer and direct binding of XBP1 to promoter was not detected by ChIP. However, a search of the neighboring chromosomal DNA for XBP1 consensus sites revealed a sequence ~6 kb downstream of *ifnb1* that does bind XBP1, IRF3, and CBP during concomitant ER stress and LPS signaling and appears to be an ER stress-responsive enhancer element (Zeng et al., 2010). Interestingly, LPS stimulation of macrophages undergoing a UPR resulted in increased recruitment of IRF3 and CBP to the canonical *ifnb1* enhancer/promoter. XBP1 belongs to the CREB family of transcription factors and thus may directly interact with CBP/p300 as suggested by overexpression studies with tagged constructs. Interactions between XBP1 and CBP might strengthen factor recruitment to the *ifnb1* regulatory elements (Zeng et al., 2010). However, the precise relationship between XBP1 and increased IRF3 remained unclear.

Further investigation revealed that ER stress alone was sufficient to induce nuclear localization of IRF3, in an XBP1 independent manner ER stress resulted in phosphorylation of IRF3 at S386, but LPS was required for S396 phosphorylation (and thus presumed oligomerization, CBP-association, DNA-binding, and transactivation; Chen et al., 2008; Liu et al., 2012). How ER stress leads to IRF3 initial phosphorylation and nuclear translocation appears to depend upon the type of ER stress. ER stress that involves calcium dysregulation (thapsigargin treatment, calcium ionophore A23187, oxygen–glucose deprivation) appears to depend upon STING and TBK1. Through unclear mechanisms, induction of ER stress mobilizes the ER-resident STING, inducing its co-localization with TBK1 (Liu et al., 2012). Another group working in an alcohol steatosis model found that alcohol induced both XBP1 splicing and IRF3 phosphorylation in a STING-dependent manner, though the relationship between ER stress and STING activation was not directly assessed (Petrasek et al., 2013). Other forms of UPR induction (e.g., tunicamycin treatment) activate IRF3 in a STING-independent, but S1/S2 protease inhibitor sensitive process (Liu et al., 2012). This work emphasizes that not all types of UPR induction triggers the same pathways.

These results raise some intriguing questions. If the UPR activates NF-κB, AP1, and nuclear translocation of IRF3, why then is it such a poor inducer of IFN-β? The answer may lie in the particular requirements for full IRF3 activity. Given the enabling role for phosphorylation at S386, ER stress may synergize with PRR activation of IRF3 by increasing S396 phosphorylation, but the PRR signal remains indispensible. If the UPR and PRR agonists cooperate in IRF3 activation, why are certain IRF3-regulated genes not synergistically induced (e.g., RANTES)? UPR transcription factor binding sites have been found in gene regulator elements for IL-6, TNF-α, IFN-β (XBP1 binding), and IL-23 (CHOP binding; Goodall et al., 2010; Martinon et al., 2010; Zeng et al., 2010). The restriction in IRF3-regulated genes may relate to lack of binding sites for UPR-transcription factors; however this hypothesis would need to be confirmed experimentally. A requirement for both PRR stimulus and UPR-factor binding site might preserve specificity for situations involving both infection and stress, and also underlie the observed synergistic (rather than additive) degree of cytokine enhancement.

### SELECTIVE UPR PATHWAY ACTIVATION IN INNATE IMMUNE SENSING

The UPR stimulates cytokine production directly and dramatically synergizes with PRR signaling to augment IFN and other inflammatory mediators. It has become apparent that pathogen triggered PRRs may also engage UPR molecules or parts of UPR pathways to induce cytokine production, independently of a global UPR. Indeed multiple examples have been described where PRR engagement actually suppresses canonical UPR activity. For instance, LPS suppresses ATF6 and PERK pathway signaling, as evident by decreased ATF6 cleavage, BiP, ATF4, and CHOP induction (Woo et al., 2009). Yet engagement of TLR2 and TLR4 (but not TLR3, 7, or 9) in macrophages stimulates IRE1-dependent XBP1 splicing (Martinon et al., 2010). It was not clear whether the TLR specificity reflected endosomal vs. surface locations, cell type, or specific signaling pathways. Traditional XBP1 targets such as ERdj4 were not induced by TLR engagement, yet the spliced XBP1 was essential for optimal TLR stimulation of multiple cytokines and inflammatory mediators, including IL-6, ISG15, TNF-α, IFN-β, and COX2. TLR mediated IRE1 activation and XBP1 splicing appears to proceed through the NADPH oxidase NOX2 pathway (Martinon et al., 2010).

As another example of selective pathway engagement, cytosolic stimulation of PKR by dsRNA results in eIF2α phosphorylation, selective ATF4 translation, and GADD34 induction. However, in comparison to the effect of GADD34 during the UPR, polyI:C-stimulated global translational inhibition was not relieved upon the dephosphorylation of eIF2α. However, certain transcripts, including those for IL-6, IFN-β, and PKR itself continue to be translated in a GADD34 dependent manner (Clavarino et al., 2012b). Although this PKR pathway induces CHOP at the transcriptional level, CHOP translation is inhibited. As an example of how this pathway affects viral responses, Chikungunya virus-induced IFN-β was severely compromised in the absence of GADD34 (Clavarino et al., 2012a). Interestingly, engagement of this pathway by both cytosolic polyI:C and soluble polyI:C (signaling through TLR3) largely depended upon the adaptor signaling molecule TRIF. The authors propose that the TRIF–PKR–GADD34 pathway might work in parallel with the MDA-5 pathway for dsRNA sensing.

Cholera toxin sensing also coopts another specific pathway within the UPR. The cholera toxin A (CTA) protein transits into the ER and activates the RNase portion of IRE-1 to initiate RIDD. However, CTA does not activate the ATF6 or PERK pathways. RIG-I senses the small RNA fragments generated by RIDD leading to activation of NF-κB and inflammatory cytokine production. This signaling pathway is both PERK and XBP1 independent. IRE1 endonuclease activity was also required for full induction of IL-6 and IL-8 by Shiga toxin and SV40 virus, which both transit to the ER (Cho et al., 2013).

These examples of selective engagement of XBP1 splicing, GADD34 induction and IRE1 RIDD activity by immune sensors of microbial infection reveal that UPR molecules may be coopted without engagement of the full UPR. Thus the infected cell may utilize stress-signaling pathways without engaging unwanted consequences of the UPR such as apoptosis. This activation of UPR-related molecules and limited UPR pathways by PRR engagement has led to the proposal of a distinct “Microbial Stress Response” (Claudio et al., 2013). However such a response would not necessarily be exclusive of a role for the UPR in initiating or supporting inflammation.

### THE YANG FOR THIS YIN

The multiple pathways by which the UPR supports inflammation, and more specifically IFN production, would render it a potentially hazardous response for a virus to induce, even in support of its own replication. However, as an evolutionary counter, viruses have also coopted the UPR to suppress the antiviral program. For instance, activation of the PERK pathway by VSV and HCV results in phosphorylation and consequent ubiquitination of the IFNAR1 chain, decreasing IFN responsiveness. PERK^-^^/^^-^ cells were actually more resistant to VSV infection (Liu et al., 2009). Coronavirus 3a protein sabotages IFNAR signaling in a similar fashion (Minakshi et al., 2009). HCV antagonizes IFN-β production via CHOP and subsequent autophagy activation (Ke and Chen, 2011). Continued study of UPR–pathogen–cytokine relationships is likely to reveal further layers of complexity.

## CONCLUSION AND FUTURE PERSPECTIVES

**FIGURE 2:**
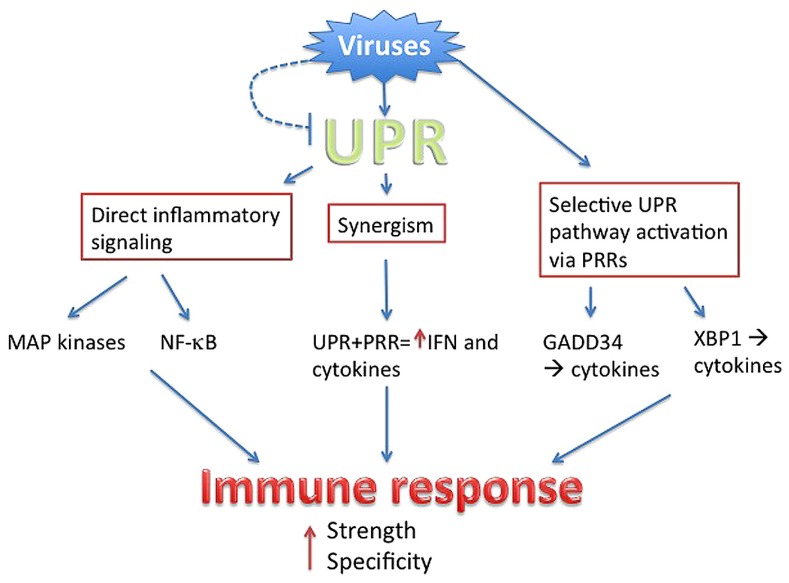
**Summary.** Viruses directly or indirectly trigger the UPR, but have evolved to antagonize parts of the UPR (dashed line). Through PRR signaling, viruses also mobilize specific parts of UPR pathways. The UPR intersects with inflammatory activation through multiple mechanisms, ultimately boosting the strength of anti-viral IFN and cytokine production. Sensing of the virus via UPR and UPR-related pathways provides context, ensuring specificity.

Even as viruses utilize the host UPR to enhance virus production and host cell survival, the invoked UPR in turn has the potential to augment anti-viral responses. Multiple mechanisms intertwining the UPR and inflammatory/IFN responses have been described, from direct activation of cytokine transcription factors to UPR–PRR synergy and selective UPR pathway induction in a “microbial stress response” (Claudio et al., 2013). These pathways are not necessarily exclusive, but may cooperate to ultimately boost the immune response beyond the threshold needed to counteract viral subterfuge (**Figure 2**).

Several reports suggest this proposed danger signal is not just limited to the infected cell, but may be transmitted to neighboring cells. ER stress can lead to the surface expression of calreticulin, thus enhancing inflammatory cytokine production and phagocytosis of the stressed (infected) cell (Peters and Raghavan, 2011). ER stressed tumor cells can “transmit” stress to macrophages by some undefined soluble factor, resulting in macrophage UPR and cytokine production (Mahadevan et al., 2011). Might this also be true for ER stressed infected cells? The effect of infection-triggered UPR on non-infected cells adds another interesting dimension for future potential investigations.

While the model for UPR as virus sensing “danger” signal is attractive, current evidence for relevance during viral infection is limited. XBP1 has been reported to be critical for control of VSV by dendritic cells, related to elaboration of type I IFN (Hu et al., 2011). Neonatal GADD34^-^^/^^-^ mice demonstrated greater susceptibility to infection with Chikungunya virus (Clavarino et al., 2012a). There is more experimental support for the interaction of pathogens, UPR, and cytokine production from the bacterial literature. XBP1 is critical for control of *Francisella* infection in mice (Martinon et al., 2010). The UPR also apparently plays a role in macrophage immune sensing of intracellular *Brucella* infection: specifically the IRE1 pathway promotes IL-6 production (de Jong et al., 2013). More work is clearly needed to elucidate the role of the UPR in viral sensing and cytokine production in defined *in vitro* and *in vivo* models.

## Conflict of Interest Statement

The author declares that the research was conducted in the absence of any commercial or financial relationships that could be construed as a potential conflict of interest.
